# Stent Embolization in Spontaneous Coronary Artery Dissection and Its Deployment at the Right Radial Artery

**DOI:** 10.7759/cureus.14812

**Published:** 2021-05-03

**Authors:** Akash Batta, Sourabh Agstam, Soumitra Ghosh, Basant Kumar

**Affiliations:** 1 Department of Cardiology, Post Graduate Institute of Medical Education & Research, Chandigarh, IND; 2 Department of Cardiology, Vardhman Mahavir Medical College and Safdarjung Hospital, New Delhi, IND

**Keywords:** spontaneous coronary artery dissection, acute coronary syndrome, stent embolization.

## Abstract

Spontaneous coronary artery dissection (SCAD) is an unusual but important cause of acute coronary syndrome and is often underdiagnosed. The first clue to the diagnosis is the angiographic appearance of the lesion, and, in certain cases, intravascular imaging is needed to confirm it. Conservative management is the preferred treatment strategy for the majority of cases. However, revascularization is needed in the presence of high-risk features, including hemodynamic instability, ongoing ischemia, and left main dissection.

We report a case of a 43-year-old man who presented with acute inferior wall myocardial infarction. Angiogram revealed SCAD of the right coronary artery (RCA). In view of ongoing chest pain, we proceeded with direct stenting. However, during the stent delivery, the stent got embolized and laid unexpanded in the proximal RCA. The stent was successfully retrieved and was deployed at the right radial artery. Subsequently, after the troubleshoot, we again secured wire access across the RCA, and this time after pre-dilatation, successful stenting across the SCAD segment was achieved.

Percutaneous coronary intervention (PCI) in SCAD is technically challenging with lower success and higher complication rates compared to atherosclerotic disease. Stent embolization is a potential complication during PCI of SCAD and to the best of our knowledge has never been reported before. Though, in general, the SCAD lesion is soft and one may proceed with direct stenting with long stents, occasionally adequate pre-dilatation may be necessary in order to facilitate the smooth passage of stent across the lesion.

Though stent embolization in SCAD is a rare event, the operator must be aware of such a possibility and the potential bailout strategies if faced with such a scenario.

## Introduction

Spontaneous coronary artery dissection (SCAD) is an underrecognized cause of myocardial infarction (MI) and sudden cardiac death typically affecting young and middle-aged women [1]. Overall, it accounts for 0.1% to 0.4% of all cases of acute coronary syndrome (ACS) [2]. It is characterized by dissection and hematoma formation within the media of coronary arteries with or without intimal dissection with resultant luminal narrowing and obstruction causing myocardial ischemia [3]. It is non-iatrogenic and not associated with atherosclerosis.

Recognition of SCAD is important, as management differs substantially from typical ACS. Conservative management is the preferred initial treatment strategy. However, revascularization is considered in certain high-risk groups including ongoing chest pain or objective evidence of ischemia, hemodynamic instability, or left main dissection [4].

Coronary stent embolization is a rare and potentially lethal complication of percutaneous coronary intervention (PCI). The index case had SCAD of the right coronary artery (RCA), and during PCI, stent embolization occurred. We will discuss the potential factors in our case that lead to stent embolization and how we managed it.

## Case presentation

**Figure 1:**
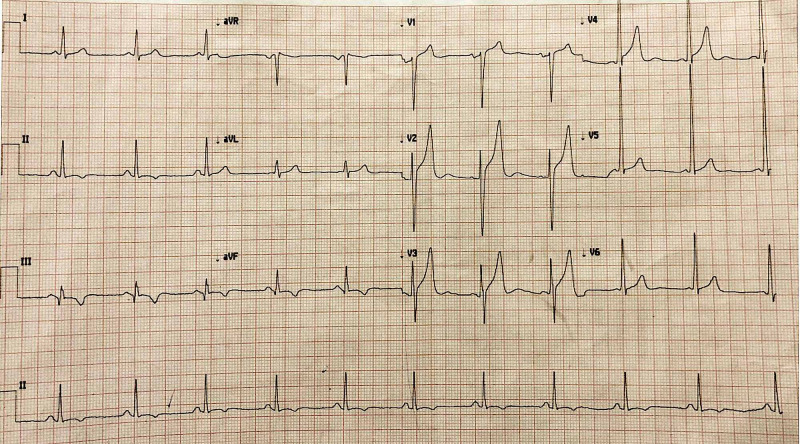
A 12-lead electrocardiogram (EKG) showing pathological Q waves and T wave inversion in II, III, and aVF leads suggestive of inferior wall myocardial infarction.

A 43-year-old male without prior comorbidities presented in the emergency room with complaints of atypical chest pain for 12 hours prior to admission. At presentation, pulse rate was 50 beats/minute, blood pressure was 110/80 mmHg, and respiratory rate was 14 breaths/minute. A 12-lead electrocardiogram (EKG) showed sinus rhythm with pathological Q waves and T wave inversion in leads III and aVF, suggestive of inferior wall MI (Figure 1). Cardiac troponin I was elevated at 3.2 ng/mL. Echocardiogram revealed hypokinesia of the inferior wall with an ejection fraction of 45%.

**Figure 2:**
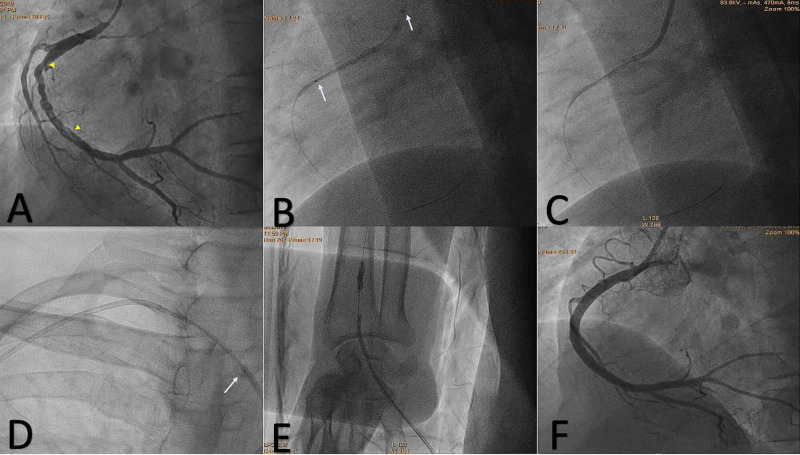
(A) SCAD involving the mid-distal RCA (arrowheads). Embolized stent (B) (arrows) laid unexpanded in the proximal RCA (C). Entire assembly being pulled out (D) and crumpled stent deployed at right radial artery (E). Final angiogram after stenting (F). SCAD, spontaneous coronary artery dissection; RCA, right coronary artery

Subsequently, an angiogram performed via right radial access revealed a normal angiogram of the left circulation and typical angiographic features of SCAD including multiple radiolucent lumens from mid to distal RCA, fitting into type 1 SCAD (Figure 2A). Because of ongoing pain and rising cardiac troponin I (7.4 ng/mL) in the index case, he was taken up for PCI to RCA after informed consent.

Judkins Right guide (JR 6F) (Medtronic, Minneapolis, MN, USA) was engaged, and the lesion was crossed with a SION blue 0.014-inch coronary wire (ASAHI Intecc Co., Aichi, Japan). After wiring the RCA, direct stenting without pre-dilatation was undertaken given the ongoing ischemia and to shorten the procedure duration. Tetrilimus™ (SMT Pvt. Ltd., Gujarat, India) 4 x 40 mm everolimus-eluting stent was taken and advanced up to the dissection flap. Further advancement of the stent was difficult, and while negotiating it through the dissection flap, the stent got embolized from the underlying balloon and laid unexpanded in the proximal RCA. Its proximal edge was seen overhanging in ascending aorta (Figures 2B, 2C).

Subsequently, to remove the embolized stent, Tazuna® (Terumo, Tokyo, Japan) 2.5 x 10 mm semi-compliant balloon was used across the distal stent edge over the coronary wire; the balloon was inflated to 16 atmospheres (atm) and was pulled to engage in distal stent struts. Once it was engaged, the tug was given at the distal stent edge and upon confirmation of its grip, the entire assembly, including the guiding catheter and the coronary wire, was pulled out through the aorta, subclavian, axillary, and brachial artery up to the radial artery (Figure 2D).

An obvious crumpling of the undeployed stent was noted. The stent along with the inflated balloon could not be pulled out of the radial artery despite multiple attempts. Therefore, the balloon was deflated and pulled back into the crumpled stent and reinflated at 16 atm. Thus, ultimately, the stent was deployed in the right radial artery as a bailout procedure (Figure 2E).

After troubleshoot, femoral access was taken and JR 6F guide was engaged to RCA. A note was made of the dissection in the proximal RCA as a result of manipulations done at the time of stent embolization. The lesion was successfully wired using SION blue 0.014-inch coronary wire. Subsequently, this time around, adequate pre-dilatation using 1.5 x 10 mm and 2.5 x 15 mm semi-compliant Tazuna balloons was performed.

**Figure 3:**
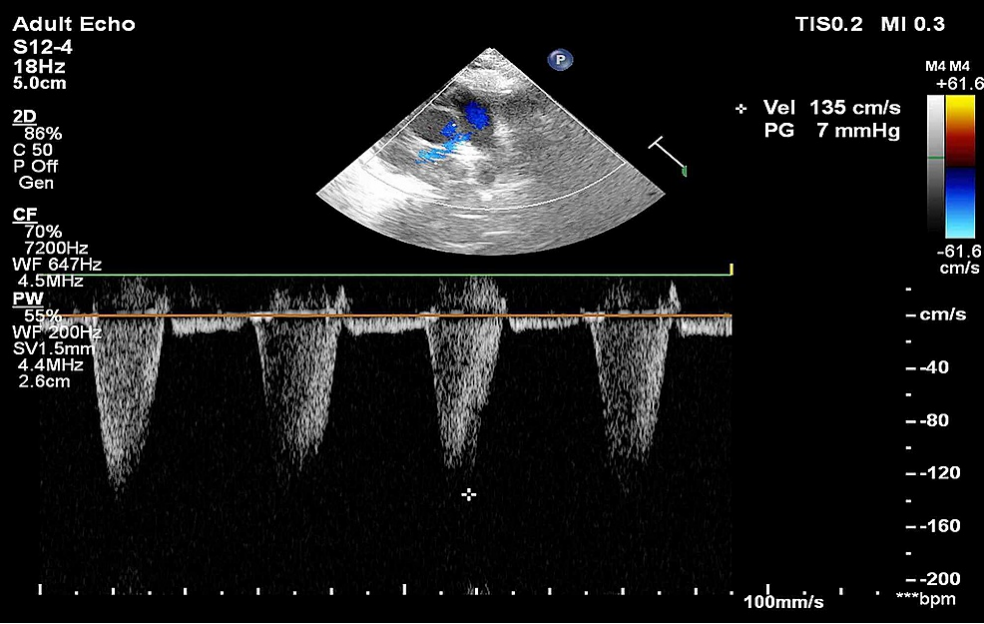
Color Doppler ultrasound of the right radial artery distal to the deployed stent shows a normal phasic pattern suggestive of a patent lumen with adequate flow.

After pre-dilatation, three overlapping sirolimus-eluting stents were implanted successfully: Tetrilimus 4 x 40 mm was deployed across the mid to distal RCA, Tetrilimus 4 x 24 mm in the mid-RCA, and Evermine50 (Meril, Gujarat, India) 4.5 x 32 mm from the ostial to mid-RCA. Post-dilatation was performed with a 4.5 x 15 mm non-compliant Pantera® Leo (Biotronik, Berlin, Germany) balloon from the distal to ostial RCA. The final angiogram revealed TIMI III flow across RCA (Figure 2F). After 24 hours, radial artery patency was confirmed by color Doppler ultrasonography (Figure 3).

The patient had an uneventful course and was subsequently discharged after two days. The patient is doing well at 12 months of follow-up.

## Discussion

PCI in SCAD is technically challenging with low success rates and high complication rates. The common difficulties faced by operators include difficult wiring, dissection extension, no-reflow, risk of side branch occlusion, and stent-related complications including stent thrombosis and restenosis [5]. SCAD most commonly involves the mid to distal left anterior descending artery [6]. ST-elevation MI is the commonest presentation followed by non-ST elevation MI [7].

Coronary angiography is the primary modality for diagnosis; however, it has limited ability to accurately diagnose intermural hematoma, for which intracoronary imaging modalities such as optical coherence tomography (OCT) or intravascular ultrasound (IVUS) are required [8]. In the majority of the patients, the dissected segment heals spontaneously, and conservative management with beta-blockers usually suffices. However, a subset of patients with SCAD have a malignant course and require intervention [4].

Coronary stent embolism occurs when a coronary stent is unintentionally dislodged into the coronary or systemic circulation. With the advent of balloon pre-mounted stents with lower crossing profiles and the improvements in the stent delivery system, the incidence has reduced significantly. The common scenarios that increase the risk of stent embolization include severely calcified lesions, significant vessel angulation, manual handling of the stent, prior deployment of a proximal stent, direct stenting in a critical lesion, and poor support of the guidewire or the guiding catheter [9]. Adequate bed preparation with pre-dilatation of the coronary lesion decreases the chance of stent embolism [9].

Various techniques have been described for the management of an embolized stent. These include crushing the embolized stent with the help of a balloon or another stent against the vessel wall, use of a snare for stent retrieval, passing another wire beyond the embolized stent and then twisting the two wires to engage in stent struts followed by removal, and, as in our case, passing a small balloon distal to the embolized stent followed by its inflation and subsequent removal of the entire assembly [9]. Deployment of the embolized stent at an unintended coronary or peripheral site with the help of a balloon or second stent is another option.

The factors that could have contributed to stent embolization in this index case are inadequate bed preparation through pre-dilatation and poor guiding support while negotiating the stent through the proximal RCA angulation. IVUS was not used, which could have guided us and lead to a more meticulous approach during PCI.

The index case has a limitation that intravascular imaging with IVUS or OCT could not be performed because of unavailability at that point in time, which would have made us more confident in our diagnoses and provided us with intraprocedural guidance.

## Conclusions

Though stent embolization in SCAD is a rare event, the operator must be aware of such a possibility and the potential bailout strategies if faced with such a scenario.
